# The survival and the long-term trends of patients with gastric cancer in Shanghai, China

**DOI:** 10.1186/1471-2407-14-300

**Published:** 2014-04-29

**Authors:** Leizhen Zheng, Chunxiao Wu, Pan Xi, Meiling Zhu, Li Zhang, Siyu Chen, Xiaoping Li, Jianchun Gu, Ying Zheng

**Affiliations:** 1 Department of Oncology, Xin Hua Hospital affiliated to Shanghai Jiaotong University School of Medicine, Shanghai, China; 2 Shanghai Municipal Center for Disease Control and Prevention, Shanghai, China

## Abstract

**Background:**

Gastric cancer remains a major health issue and a leading cause of death worldwide. This study presented a long-term survival data of gastric cancer registered in Shanghai of China from 1972–2003, with aims to describe the trends as well as the age, sex, stage and tumor sites specific characteristics.

**Methods:**

The main source of information on cancer cases was the notification card sending to the registry. The residential status of cancer cases was confirmed by home-visits. The methods of follow-up have been a mixture of both active and passive ones.

**Results:**

We observed an increased trend of survival probability during the last decades. Patients diagnosed during 1972–1976 had a 5-years relative survival rate at 12% for males and 11% for females, respectively, which had dramatically increased to 30% for male and 32% for female patients respectively during the period of 2002–2003. Among the patients diagnosed in 2002–2003, the overall survival probability declined with patient’s age at the time of diagnosis. The lowest survival rate was observed among the oldest group, with the median survival time of 0.8 years. Patients diagnosed with stage I had a higher relative survival rate. Patients with cardia cancer had the worst prognosis, with the 5-year relative survival rate of 29%.

**Conclusions:**

The survival probability of patients with gastric cancer in Shanghai has improved significantly during the last decades. Age, stage and site of tumor have an impact on prognosis.

## Background

Gastric cancer is one of the most common cancers worldwide, accounting for about 8% of all new cancer cases [1]. In recent decades, the morbidity and mortality of gastric cancer have fallen down dramatically, with the decline first taking place in the countries with low incidence while the decline in the regions with high incidence was relatively slow [2-4]. Compared with the steady decrease in the United States and European countries, the incidence of gastric cancer remains high in the Far East areas, where up to 100 cases per 100000 populations are reported annually [4]. Despite an overall decrease of incident gastric cancer has been observed in China, the decline was less dramatic than other countries and even there was an increase in the youngest and oldest groups [5].

In recent years, prognosis of patients with gastric cancer has improved significantly [6]. Like other health indices, information on survival statistics is an important component in monitoring cancer control activities, which may suggest possible reasons for the variations and provide targets for the improvement towards them. Based on the registry database of gastric cancer in Shanghai of China since 1970s, we performed the current descriptive study. The aim of this study is to present the survival data and to illuminate relevant trends as well as the age, sex, stage of disease and tumor sites specific characteristics.

## Methods

Shanghai is situated on the bank of the Yangtz River Delta of Eastern China, which is the largest city in China and the eighth largest city in the world, with a population of 14.0 million (2010 year-end registered population). It includes urban and suburb areas, where the urban covers 9 districts locating in the center of the municipality, and the suburb covers the other 9 districts. The data of gastric cancer for this study were derived from the Shanghai Cancer Registry Center (SCRC), compiled by the Shanghai Center for Disease Control and Prevention (CDC). The SCRC was established in the 1960s, which has been contributing data to the quinquennial IARC publication Cancer Incidence in Five Continents since Vol. IV [7]. The main source of information on cancer cases was the notification card consisting of basic information for cancer registration that was sent to the SCRC. The residential status of cancer cases was confirmed by home-visits [7]. We obtained the follow-up data of gastric cancer patients diagnosed in 2002–2003 for survival trend analysis, as well as previous published survival data of the other four periods (1972–1976, 1980–1984, 1988–1991, and 1992–1995) which were referred to the “Cancer incidence, mortality and survival rates in urban Shanghai (1973–2000)” [8] and “Cancer survival in Shanghai, China (1992–1995) [9]. We identified the gastric cancer cases using the 10th Revision of the International Classification of Diseases (code C16) [10]. The cancer registry has been using AJCC/UICC TNM staging system in combination with the clinical stages classification system (I-IV).

Patients reported to SCRC were followed up for confirmed diagnosis and survival. The methods of follow-up have been a mixture of both active and passive ones. With passive follow-up, information on death is routinely obtained from the death certificates in the vital statistical section of Shanghai CDC. Through this approach, patients whose death information has not been received may be considered to be “alive” until that point of time. Furthermore, the mortality data are periodically matched with the incident database of cancers. The vital status of the unmatched incident cases is then verified by home visits or postal/telephone enquiries. Active follow-up is necessary in the absence of reliable health information. Reports of deaths from hospitals, living status from the community, or loss to follow-up were updated until December 31, 2010. The censor was defined as still alive at the closing date, or lost to follow-up or died by other causes other than gastric cancer. The survival probability was estimated using random censoring data. In the study, a total of 61140 gastric cancer cases were included for the above-mentioned five periods (Additional file 1: Table S1), among which 10909 cases were registered during the period of 2002 to 2003. The study was approved by the Ethical Review Committee at Shanghai Municipal CDC [11].

Both observed and relative survival probabilities were estimated. Life tables were constructed to calculate the cumulative probability of survival at time t_i+1_ from the conditional probabilities of survival during consecutive intervals of follow-up time up to and including t_i+1_. Relative survival is a measure of net survival calculated by comparing observed survival with expected survival from a comparable set of people that do not have cancer. It is used to measure the excess mortality that is associated with a cancer diagnosis, and is designed to exclude the effect arising from different background mortalities. The relative survival (R_i_) for a group of patients at the end of an interval beginning at time t_i_ was defined as $R_{i}=\frac{S_{i}}{S_{i}^{*}}$, where S_i_ was the absolute survival for subjects with a gastric cancer and S_i_^*^ was the expected survival of a group of individuals with the same demographic characteristics (age, and sex, etc.) who were at risk of death only from causes other than the cancer under study. Chi-Square test was used to compare the distribution between males and females. Furthermore, Log rank test was used to compare the survival rates with 95% confidence interval (CI). Written informed consent was obtained from the patient for the publication of this report and any accompanying images.

## Results

As showed in Table 1, there were 10909 newly diagnosed gastric cancer cases reported during 2002–2003, including 47.1% living in the urban and 52.9% living in the suburb. Among them, there were 7038 (64.5%) males and 3871 (35.5%) females. Patients aged 65–84 years accounted for more than 58% of all cases.The proportion of patients being classified as stage I to IV was 5.5%, 9.9%, 12.4%, and 13.8% respectively, while 58.4% of cases were reported with “unknown stage”. The gender difference of tumor sites was significant (χ^2^ = 79.41, *P <* 0.001). Malignant neoplasm of pyloric antrum account for 22.3% and 4796 (44.0%) cases were reported with unspecified sites. At the time of the last follow-up (December 31, 2010), 8365 (76.7%) patients died, 2312 (21.2%) patients were alive, and 232 (2.1%) cases were lost to follow up.

**Table 1:** General information of patients with gastric cancer during 2002–2003 in Shanghai, China

**Variables**		**Total**	**Male**	**Female**	**χ**^**2#**^	***P***
Area						
	Urban	5139 (47.1)	3270 (46.5)	1869 (48.3)	3.32	0.068
	Suburb	5770 (52.9)	3768 (53.5)	2002 (51.7)		
Age-group						
	15-	107 (1.0)	45 (0.6)	62 (1.6)	125.18	<0.001
	35-	541 (5.0)	292 (4.1)	249 (6.4)		
	45-	1625 (14.9)	1032 (14.7)	593 (15.3)		
	55-	1720 (15.8)	1220 (17.3)	500 (12.9)		
	65-	3545 (32.5)	2367 (33.6)	1178 (30.4)		
	75-	2881 (26.4)	1828 (26.0)	1053 (27.2)		
	85+	490 (4.5)	254 (3.6)	236 (6.1)		
Tumor site*						
	C16.0	1371 (12.6)	1005 (14.3)	366 (9.5)	79.41	<0.001
	C16.1	171 (1.6)	119 (1.7)	52 (1.3)		
	C16.2	873 (8.0)	536 (7.6)	337 (8.7)		
	C16.3	2436 (22.3)	1545 (22.0)	891 (23)		
	C16.4	106 (1.0)	67 (1.0)	39 (1.0)		
	C16.5	758 (6.9)	527 (7.5)	231 (6)		
	C16.6	62 (0.6)	30 (0.4)	32 (0.8)		
	C16.8	336 (3.1)	222 (3.2)	114 (2.9)		
	C16.9	4796 (44.0)	2987 (42.4)	1809 (46.7)		
Stage						
	I	601 (5.5)	394 (5.6)	207 (5.3)	8.371	0.079
	II	1085 (9.9)	721 (10.2)	364 (9.4)		
	III	1353 (12.4)	902 (12.8)	451 (11.7)		
	IV	1504 (13.8)	982 (14.0)	522 (13.5)		
	Unknown	6366 (58.4)	4039 (57.4)	2327 (60.1)		
Vital status						
	Died	8365 (76.7)	5454 (77.5)	2911 (75.2)	7.96	0.019
	Survival	2312 (21.2)	1445 (20.5)	867 (22.4)		
	Lost to follow up	232 (2.1)	139 (2.0)	93 (2.4)		

*C16.0, Malignant neoplasm of cardia; C16.1, Malignant neoplasm of fundus of stomach; C16.2, Malignant neoplasm of body of stomach; C16.3, Malignant neoplasm of pyloric antrum; C16.4, Malignant neoplasm of pylorus; C16.5, Malignant neoplasm of lesser curvature of stomach, unspecified; C16.6, Malignant neoplasm of greater curvature of stomach, unspecified; C16.8, Malignant neoplasm of overlapping sites of stomach; C16.9, Malignant neoplasm of stomach, unspecified.

^#^Chi-Square test was used to compare the distribution between males and females.

### Overall survival rate

Among gastric cancer patients diagnosed during the period 2002–2003 in Shanghai, the 1-year observed survival rate was 51% for male and 52% for female, respectively. The 5-years observed survival rate decreased to 25% for male and 27% for female, respectively. The 1-year relative survival rate was 54% for male and 56% for female patients, respectively. The 5-years relative survival rate was 30% for male and 32% for female patients, respectively. The median survival time was 1.09 years. Patients living in the urban had slightly higher survival rate compared with the patients in the suburb (Table 2).

**Table 2:** Survival rate of patients with gastric cancer in different regions during 2002–2003 in Shanghai, China

**Area**	**Sex**	**N**	**Observed survival rate [% (95% CI)]**					**Relative survival rate [% (95% CI)]**					**Median survival (years)**
			**1 year**	**2 years**	**3 years**	**4 years**	**5 years**	**1 year**	**2 years**	**3 years**	**4 years**	**5 years**	
	Total	10909	51 (50–52)	37 (36–38)	31 (30–32)	28 (27–29)	26 (25–27)	55 (54–56)	41 (40–42)	35 (34–36)	32 (31–33)	30 (29–31)	1.09
Whole city	Male	7038	51 (50–52)	37 (36–38)	30 (29–31)	27 (26–28)	25 (24–26)	54 (53–55)	40 (39–41)	34 (33–35)	31 (30–32)	30 (29–31)	1.06
	Female	3871	52 (50–54)	38 (36–40)	32 (31–34)	30 (29–31)	27 (26–28)	56 (54–58)	42 (40–44)	37 (36–39)	34 (33–36)	32 (31–34)	1.14
	χ^2^*		1.05	2.14	6.29	10.09	6.42	-	-	-	-	-	
	P*		0.305	0.144	0.012	0.001	0.011	-	-	-	-	-	
Urban	Total	5139	52 (51–53)	38 (37–39)	32 (31–33)	28 (27–29)	26 (25–27)	56 (55–57)	42 (41–43)	36 (35–37)	33 (32–34)	31 (30–32)	1.13
	Male	3270	51 (49–53)	37 (35–39)	30 (28–32)	27 (25–29)	25 (24–27)	55 (53–57)	41 (39–43)	35 (33–37)	32 (30–34)	31 (29–33)	1.07
	Female	1869	53 (51–55)	40 (38–42)	34 (32–36)	31 (29–33)	28 (26–30)	58 (56–60)	45 (43–47)	39 (37–41)	36 (34–38)	33 (31–35)	1.24
	χ^2^*		2.02	6.67	6.29	8.65	3.77	-	-	-	-	-	
	*P*		0.155	0.010	0.012	0.003	0.052	-	-	-	-	-	
Suburb	Total	5770	51 (50–52)	36 (35–37)	30 (29–31)	27 (26–28)	26 (25–27)	54 (53–55)	39 (38–40)	33 (32–34)	31 (30–32)	30 (29–31)	1.05
	Male	3768	51 (49–53)	37 (35–39)	30 (29–32)	27 (26–28)	25 (24–26)	54 (52–56)	40 (38–42)	33 (32–35)	31 (30–33)	29 (28–31)	1.05
	Female	2002	51 (49–53)	36 (34–38)	31 (29–33)	29 (27–31)	27 (25–29)	55 (53–57)	39 (37–41)	35 (33–37)	33 (31–35)	31 (29–33)	1.05
	χ^2^*		0.01	0.21	1.11	2.48	2.70	-	-	-	-	-	
	P*		0.954	0.645	0.292	0.115	0.100	-	-	-	-	-	

*Log rank test was used to compare the survival rates between males and females.

### Age-specific survival rate

Table 3 showed the survival rate of gastric cancer at different ages. We excluded the cases younger than 35 years or older than 85 years for survival analysis because of insufficient number of cases at these age groups. In general, the overall survival probability declined with the increase of patient’s age at the time of diagnosis. The lowest survival rate was observed among the oldest group (75–84 years), with the median survival time of 0.8 years. The age related trend of survival rate was different between male and female patients. For male patients, the youngest group (35–44 years) had the highest survival rate with the longest median survival time of 2.3 years. However, the survival probability at 35–44 years age group was worse than those aged 45–64 years among female gastric patients (Table 3).

**Table 3:** Survival rate of patients with gastric cancer at different ages during 2002–2003 in Shanghai, China

**Age (years)**	**Sex**	**N**	**Observed survival rate [% (95% CI)]**					**Relative survival rate [% (95% CI)]**					**Median survival (years)**
			**1 year**	**2 years**	**3 years**	**4 years**	**5 years**	**1 year**	**2 years**	**3 years**	**4 years**	**5 years**	
35-44	Male	292	66 (60–71)	52 (46–58)	46 (40–52)	44 (38–50)	43 (37–49)	66 (60–72)	52 (46–58)	47 (41–53)	45 (39–51)	44 (38–50)	2.3
	Female	249	62 (56–68)	51 (45–57)	43 (37–49)	40 (34–46)	39 (33–45)	62 (56–68)	51 (45–57)	44 (38–51)	40 (34–47)	40 (34–47)	2.1
45-54	Male	1032	66 (63–69)	52 (49–55)	45 (42–48)	42 (39–45)	41 (38–44)	66 (63–69)	52 (49–55)	46 (43–49)	44 (41–47)	43 (40–46)	2.3
	Female	593	67 (63–71)	54 (50–58)	48 (44–52)	45 (41–49)	43 (39–47)	67 (63–71)	54 (50–58)	49 (45–53)	46 (42–50)	44 (40–48)	2.7
55-64	Male	1220	59 (56–62)	45 (42–48)	38 (35–41)	35 (32–38)	33 (30–36)	60 (57–63)	46 (43–49)	40 (37–43)	38 (35–41)	37 (34–40)	1.7
	Female	500	61 (57–65)	48 (44–52)	42 (38–46)	41 (37–45)	38 (34–42)	61 (57–65)	48 (44–53)	44 (40–49)	42 (38–47)	41 (37–46)	1.8
65-74	Male	2367	52 (50–54)	37 (35–39)	30 (28–32)	26 (24–28)	24 (22–26)	54 (52–56)	40 (38–42)	34 (32–36)	33 (31–35)	35 (33–37)	1.1
	Female	1178	56 (53–59)	41 (38–44)	34 (31–37)	31 (28–34)	28 (25–31)	56 (53–59)	43 (40–46)	37 (34–40)	36 (33–39)	35 (32–38)	1.4
75-84	Male	1828	37 (35–39)	23 (21–25)	17 (15–19)	14 (12–16)	12 (11–14)	40 (38–42)	29 (27–31)	26 (24–28)	28 (26–30)	34 (32–36)	0.8
	Female	1053	38 (35–41)	23 (21–26)	17 (15–19)	15 (13–17)	13 (11–15)	40 (37–43)	26 (23–29)	23 (21–26)	25 (22–28)	27 (24–30)	0.8

### Stage of disease and survival rate

Patients were classified into different clinical stages (I-IV) according to AJCC /UICC TNM staging system, where the lower stage indicated the early clinical phase of the disease. Based on the survival data from SCRC, it was obvious that patients diagnosed with lower stages had significantly better prognosis. The 5-years relative survival rate was over 80% among patients at the stage I. However, it dramatically decreased to 10% when patients were diagnosed at stage IV. The 5-years relative survival rate of males is slightly higher than that of females except for those diagnosed at stage IV(Table 4).

**Table 4:** Survival rate of patients with gastric cancer in different stages during 2002–2003 in Shanghai, China

**Stage**	**Sex**	**N**	**Observed survival rate [% (95% CI)]**					**Relative survival rate [% (95% CI)]**					**Median survival (years)**
			**1 year**	**2 years**	**3 years**	**4 years**	**5 years**	**1 year**	**2 years**	**3 years**	**4 years**	**5 years**	
I	Male	394	88 (84–91)	80 (76–84)	73 (68–77)	69 (64–73)	68 (63–73)	91 (88–94)	85 (81–88)	83 (79–87)	84 (80–88)	88 (85–91)	>5
	Female	207	90 (85–94)	80 (74–85)	74 (67–80)	71 (64–77)	67 (60–73)	93 (89–96)	85 (79–90)	81 (75–86)	82 (76–87)	81 (75–86)	>5
II	Male	721	79 (76–82)	64 (60–67)	55 (51–59)	51 (47–55)	48 (44–52)	81 (78–84)	69 (65–72)	63 (59–67)	63 (59–67)	64 (60–68)	4.38
	Female	364	78 (73–82)	66 (61–71)	58 (53–63)	53 (48–58)	50 (45–55)	81 (77–85)	70 (65–75)	64 (59–69)	61 (56–66)	61 (56–66)	4.91
III	Male	902	59 (56–62)	41 (38–44)	31 (28–34)	27 (24–30)	25 (22–28)	62 (59–65)	45 (42–48)	36 (33–39)	34 (31–37)	35 (32–38)	1.51
	Female	451	62 (57–66)	42 (37–47)	35 (31–40)	31 (27–36)	29 (25–33)	64 (60–69)	44 (39–49)	39 (35–44)	36 (32–41)	36 (32–41)	1.58
IV	Male	982	27 (24–30)	15 (13–17)	11 (9–13)	8 (6–10)	7 (6–9)	29 (26–32)	17 (15–20)	13 (11–15)	11 (9–13)	10 (8–12)	0.68
	Female	522	28 (24–32)	15 (12–18)	11 (9–14)	9 (7–12)	8 (6–11)	29 (25–33)	17 (14–21)	13 (10–16)	11 (8–14)	10 (8–13)	0.69
Unknown	Male	4039	46 (44–48)	32 (31–33)	26 (25–27)	23 (22–24)	21 (20–22)	50 (49–52)	37 (36–39)	33 (32–35)	33 (32–35)	34 (33–36)	0.93
	Female	2327	48 (46–50)	34 (32–36)	29 (27–31)	27 (25–29)	24 (22–26)	54 (52–56)	40 (38–42)	36 (34–38)	36 (34–38)	36 (34–38)	0.96

### Tumor sites and survival rate

Survival rate of gastric cancer varied among different tumor sites. The worst prognosis was observed in patients with cardia cancer, as the 5-years relative survival rate was only 29% (Table 5).

**Table 5:** Survival rate of patients with gastric cancer in different tumor sites during 2002–2003 in Shanghai, China

**Tumor sites**	**Sex**	**N**	**Observed survival rate [% (95% CI)]**					**Relative survival rate [% (95% CI)]**					**Median survival (years)**
			**1 year**	**2 years**	**3 years**	**4 years**	**5 years**	**1 year**	**2 years**	**3 years**	**4 years**	**5 years**	
C16.0 Cardia	Male	1005	49 (46–52)	33 (30–36)	24 (21–27)	20 (18–23)	18 (16–21)	52 (49–55)	37 (34–40)	30 (27–33)	28 (25–31)	29 (26–32)	1.0
	Female	366	53 (48–58)	34 (29–39)	26 (22–31)	23 (19–28)	21 (17–26)	57 (52–62)	39 (34–44)	32 (27–37)	30 (25–35)	29 (24–34)	1.1
C16.1 Fundus	Male	119	53 (44–62)	43 (34–52)	38 (29–47)	31 (23–40)	29 (21–38)	55 (45–64)	47 (38–56)	44 (35–53)	40 (31–50)	41 (32–51)	1.3
	Female	52	67 (52–79)	53 (39–67)	47 (33–61)	45 (31–59)	43 (30–57)	70 (55–81)	58 (43–71)	54 (40–68)	55 (41–70)	57 (43–71)	2.5
C16.2 Body	Male	536	55 (51–59)	39 (35–43)	32 (28–36)	29 (25–33)	27 (23–31)	57 (53–61)	43 (39–47)	37 (33–41)	37 (33–41)	38 (34–42)	1.3
	Female	337	58 (53–63)	45 (40–50)	38 (33–43)	34 (29–39)	31 (26–36)	60 (55–65)	47 (42–52)	41 (36–46)	39 (34–44)	37 (32–43)	1.6
C16.3 Pyloric antrum	Male	1545	60 (58–62)	44 (42–47)	37 (35–39)	33 (31–35)	31 (29–33)	64 (62–66)	50 (48–53)	45 (42–48)	44 (42–47)	45 (43–48)	1.6
	Female	891	59 (56–62)	46 (43–49)	39 (36–42)	37 (34–40)	34 (31–37)	63 (60–66)	51 (48–54)	45 (42–48)	45 (42–48)	45 (42–48)	1.7
C16.4 Pylorus	Male	67	71 (58–81)	55 (42–67)	49 (37–61)	44 (32–57)	41 (29–54)	77 (66–87)	62 (50–74)	59 (47–72)	58 (46–70)	59 (47–72)	2.8
	Female	39	69 (52–82)	59 (42–74)	54 (38–70)	49 (33–65)	46 (30–62)	73 (55–85)	64 (47–79)	62 (45–77)	60 (42–74)	62 (45–77)	3.7
C16.5 Lesser curvature	Male	527	74 (70–78)	60 (56–64)	53 (49–57)	50 (46–54)	47 (43–51)	76 (72–80)	64 (60–68)	60 (56–64)	60 (56–64)	61 (57–65)	3.9
	Female	231	72 (66–78)	57 (50–63)	54 (47–61)	50 (43–57)	46 (39–53)	75 (69–80)	61 (54–67)	59 (52–65)	57 (51–64)	57 (51–64)	3.9
C16.6 Greater curvature	Male	30	77 (58–90)	53 (34–71)	40 (23–59)	40 (23–59)	40 (23–59)	81 (61–92)	59 (41–77)	47 (28–66)	51 (31–69)	55 (37–75)	2.2
	Female	32	71 (52–85)	52 (34–70)	48 (30–66)	48 (30–66)	48 (30–66)	78 (60–91)	58 (41–76)	57 (38–74)	60 (41–76)	65 (47–81)	2.4
C16.8 Overlapping sites	Male	222	48 (41–55)	29 (23–36)	24 (19–30)	21 (16–27)	20 (15–26)	50 (43–57)	33 (27–40)	29 (23–35)	28 (22–34)	30 (24–37)	1.0
	Female	114	61 (51–70)	44 (35–54)	37 (28–47)	34 (26–44)	32 (24–41)	63 (54–72)	47 (38–57)	42 (33–52)	41 (32–51)	40 (31–50)	1.6
C16.9 Unspecified	Male	2987	42 (40–44)	29 (27–31)	24 (22–26)	21 (20–23)	20 (19–21)	45 (43–47)	34 (32–36)	30 (28–32)	30 (28–32)	31 (29–33)	0.9
	Female	1809	43 (41–45)	30 28–32)	25 (23–27)	23 (21–25)	21 (19–23)	48 (46–50)	35 (33–37)	31 (29–33)	31 (29–33)	31 (29–33)	0.9

### Stratification analysis by age and stage

In each age group, the 5-years survival rate declined with the increase of tumor stages. For patients at stage I and II, the 5-year survival rates were higher among those aged 35–44 years (81% and 69%, respectively). For patients at stage III–IV, the 5-year survival rates were higher among those aged 45–54 years (36% and 14%, respectively) (Table 6).

**Table 6:** Survival rate of patients with gastric cancer stratified by age and stage during 2002–2003 in Shanghai, China

**Age (years)**	Stage*	**N**	**Observed survival rate [% (95% CI)]**				
			**1 year**	**2 years**	**3 years**	**4 years**	**5 years**
35-44	I	44	95 (83–99)	88 (74–95)	86 (72–94)	81 (66–91)	81 (66–91)
	II	77	89 (79–95)	76 (65–85)	72 (60–81)	69 (57–79)	69 (57–79)
	III	80	71 (60–80)	51 (40–62)	41 (30–53)	36 (26–48)	32 (22–43)
	IV	95	22 (14–32)	12 (7–21)	5 (2–12)	4 (1–11)	3 (1–9)
45-54	I	127	98 (93–100)	93 (87–97)	87 (80–92)	83 (75–89)	82 (74–88)
	II	221	89 (84–93)	74 (68–80)	68 (61–74)	61 (54–67)	59 (52–65)
	III	244	74 (68–79)	52 (46–58)	43 (37–49)	39 (33–45)	36 (30–42)
	IV	264	33 (27–39)	20 (15–25)	18 (14–23)	15 (11–20)	14 (10–19)
55-64	I	126	90 (83–94)	86 (78–91)	82 (74–88)	80 (72–86)	78 (70–85)
	II	225	80 (74–85)	70 (63–76)	62 (55–68)	59 (52–65)	56 (49–63)
	III	243	60 (54–66)	40 (34–46)	32 (26–38)	27 (22–33)	26 (21–32)
	IV	242	35 (29–41)	17 (13–22)	12 (8–17)	9 (6–14)	9 (6–14)
65-74	I	207	87 (81–91)	76 (69–82)	69 (62–75)	67 (60–73)	64 (57–70)
	II	354	75 (70–79)	61 (56–66)	52 (47–57)	49 (44–54)	45 (40–50)
	III	469	61 (56–65)	43 (38–48)	32 (28–36)	29 (25–33)	27 (23–31)
	IV	492	29 (25–33)	16 (13–20)	11 (8–14)	9 (7–12)	7 (5–10)
75-84	I	82	77 (66–85)	57 (46–68)	45 (34–56)	37 (27–48)	31 (22–42)
	II	175	70 (63–77)	50 (42–58)	36 (29–44)	31 (24–39)	26 (20–33)
	III	276	46 (40–52)	26 (21–32)	21 (16–26)	18 (14–23)	17 (13–22)
	IV	345	21 (17–26)	12 (9–16)	9 (6–13)	6 (4–9)	5 (3–8)

*Cases with unknown stages were not involved in the analysis.

### Long-term trends of survival rate

**Figure 1:**
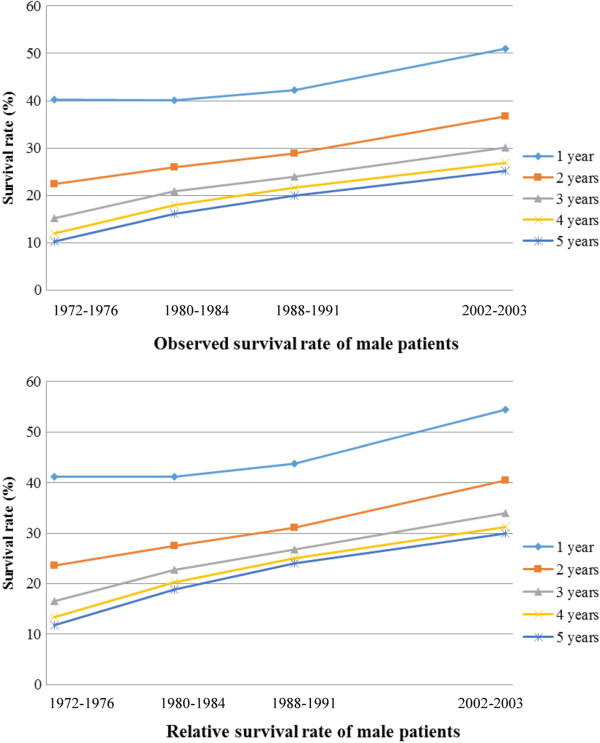
Observed and relative survival rate of male gastric cancer patients.

**Figure 2:**
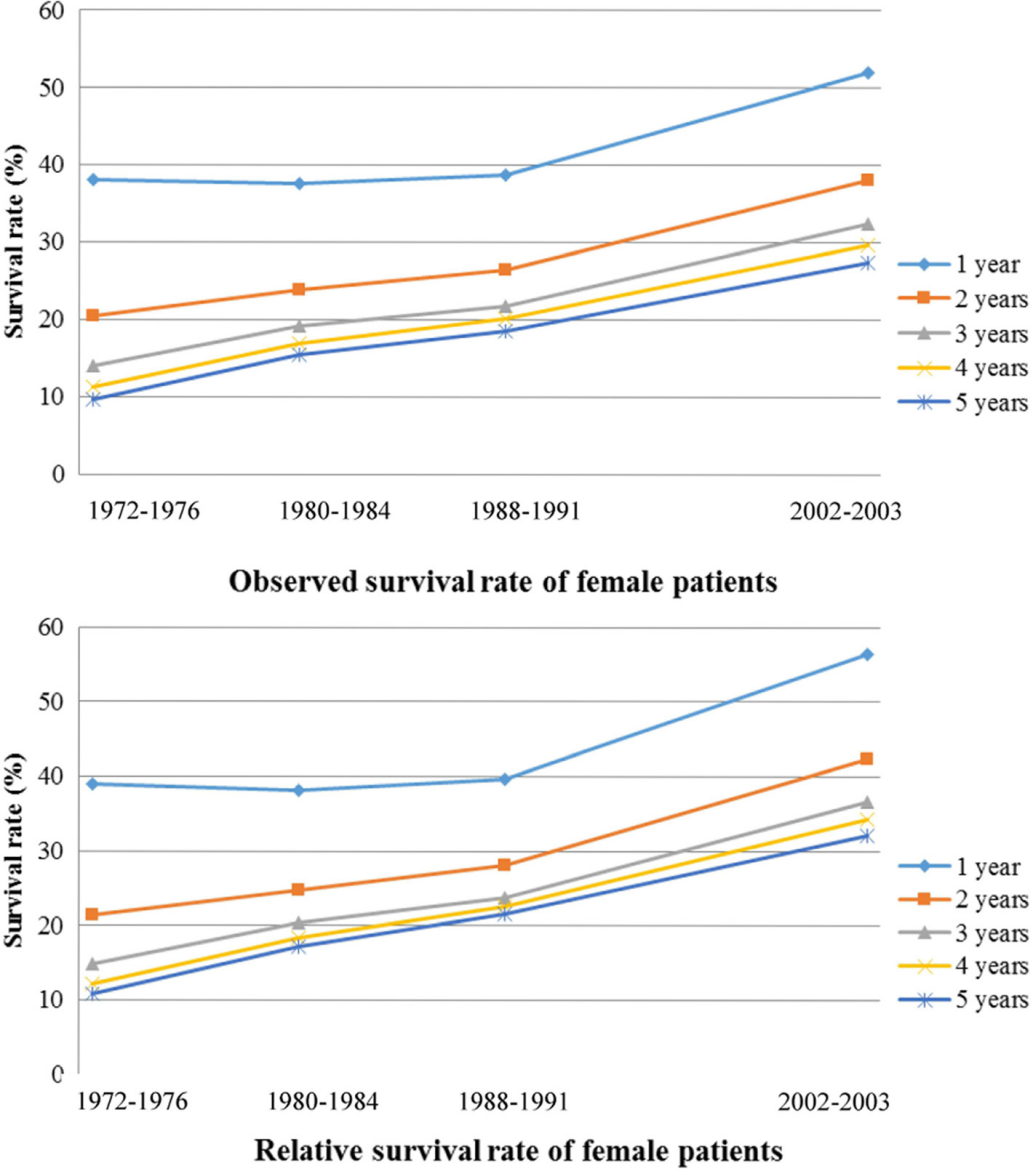
Observed and relative survival rate of female gastric cancer patients.

The trends of long-term survival data were available for patients spanning in the four time periods (1972–1976, 1980–1984, 1988–1991, and 2002–2003). An obvious increased trend of survival probability could be found during the last decades in either male or female patients (Figures 1 and 2). For example, gastric cancer patients diagnosed during 1972–1976 had a 5-years relative survival rate at 12% for males and 11% for females, respectively, which dramatically increased to 30% for male and 32% for female patients respectively during the period of 2002–2003 (Table 7).

**Table 7:** Comparison of survival rate of patients with gastric cancer from 1972 to 2003 in Shanghai, China

**Sex**	**Year**	**N**	**Observed survival rate [% (95% CI)]**					**Relative survival rate [% (95% CI)]**				
			**1 year**	**2 years**	**3 years**	**4 years**	**5 years**	**1 year**	**2 years**	**3 years**	**4 years**	**5 years**
Male	1972-1976	8287	40 (39–41)	22 (21–23)	15 (14–16)	12 (11–13)	10 (9–11)	41 (40–42)	24 (23–25)	17 (16–18)	13 (12–14)	12 (11–13)
	1980-1984	9484	40 (39–41)	26 (25-270	21 (20–23)	18 (17–19)	16 (15–17)	41 (40–42)	28 (27–29)	23 (22–24)	20 (19–21)	19 (18–20)
	1988-1991	8134	42 (41–43)	29 (28–30)	24 (23–25)	22 (21–23)	20 (19–21)	44 (43–45)	31 (30–32)	27 (26–28)	25 (24–26)	24 (23–25)
	2002-2003	7038	51 (50–52)	37 (36–38)	30 (29–31)	27 (26–28)	25 (24–26)	54 (53–55)	40 (39–41)	34 (33–35)	31 (30–32)	30 (29–31)
	χ^2*^		135.90	266.87	378.96	444.63	485.62	-	-	-	-	-
	P^*^		<0.001	<0.001	<0.001	<0.001	<0.001	-	-	-	-	-
Female	1972-1976	3860	38 (36–40)	20 (19–21)	14 (13–15)	11 (10–12)	10 (9–11)	39 (38–41)	21 (20–22)	15 (14–16)	12 (11–13)	11 (10–12)
	1980-1984	4507	38 (37–39)	24 (23–25)	19 (18–20)	17 (16–18)	15 (14–16)	38 (37–39)	25 (24–26)	20 (19–21)	18 (17–19)	17 (16–18)
	1988-1991	4224	39 (38–40)	27 (26–28)	22 (21–23)	20 (19–21)	19 (18–20)	40 (39–42)	28 (27–29)	24 (23–25)	23 (22–24)	22 (21–23)
	2002-2003	3871	52 (50–54)	38 (36–40)	32 (31–34)	30 (29–31)	27 (26–28)	56 (54–58)	42 (40–44)	37 (36–39)	34 (33–36)	32 (31–34)
	χ^2*^		154.65	277.26	308.62	344.42	316.50	-	-	-	-	-
	P^*^		<0.001	<0.001	<0.001	<0.001	<0.001	-	-	-	-	-

*Logrank test was used to compare the survival rates.

## Discussion

In this longitudinal study of gastric cancer based on Shanghai Cancer Registry database, we observed a declining trend of survival probability with the increase of patient’s age and clinical stages at the time of diagnosis. Long-term survival of gastric cancer varied among different tumor sites. The worst prognosis was observed in patients with cardia cancer. By using a long-term survival analysis on the longitudinal survival data from 1972 to 2003, we depicted that the survival probability of patients with gastric cancer in Shanghai has improved significantly during the last decades.

Gastric cancer remains a major public health issue ranking the fourth most common cancer and the second leading cause of cancer death worldwide [4]. National mortality surveys conducted in 1970s and 1990s in China, revealed an obvious cluster of geographical distribution of gastric cancer in the country, with the highest mortality mostly locating in rural areas, especially in the areas of the middle-western part of China. Despite a slight increase from the 1970s to the early 1990s, remarkable declines in gastric cancer mortality were noticed in almost the entire population in China. These declines were largely due to the dramatic improvements in the social-economic environment, lifestyle, nutrition, education, and health care system after economic reforms started decades ago [12]. Nevertheless, gastric cancer remains a cancer burden currently and be one of the key issues in cancer prevention and control strategy in China [12,13].

Data from this study revealed that the survival rate of gastric cancer patients in Shanghai was still poor. It was a little higher than that of America and some European countries [14-16], but much lower than that in Japan and Korea [17-19]. In this study, we used both observed survival rate and relative survival rate to estimate the prognosis of gastric cancer. Relative survival rate which is calculated by dividing observed survival rate by expected survival rate is designed for cancer survival studies, in order to exclude the effect resulting from different background mortalities. The 5-years relative survival rate is commonly used to monitor the progress of cancer and it reasonably indicates the average survival experience of cancer patients in a given population [20]. During the last decades over the four observation periods of 1972–1976, 1980–1984, 1988–1991, and 2002–2003, the 5-years relative survival rate of gastric cancer increased from 12% to 30% among male patients and from 11% to 32% among female patients, which seemed to be more significant than those observed in the European countries [21].

Gender difference of long-term survival of gastric cancer was not obvious in Shanghai, which was similar to the findings from other studies [22]. The prognosis of gastric cancer is closely related to the stage of disease at diagnosis. In stage I, cancer has formed in the inside lining of the mucosa (innermost layer) of the stomach wall, whereas in stage IV, cancer has spread to distant parts of the body. Early gastric cancer, whereby disease is limited to mucosa and submucosa, confers a survival rate of greater than 90% in 5 years in many centers [23]. Our study further proved that the detection of gastric cancer in the early stage is vitally important in ensuring an excellent prognosis. Every effort needs to be made to facilitate the early diagnosis of gastric cancer with aims to prolong patient’s survival time and quality of life [23]. Epidemiologic evidence supports the classification of gastric cancer into two biologically distinct disease entities, those occurring proximally (cardia) and distally (noncardia). Though carcinomas of the cardia and stomach are frequently grouped together in epidemiologic statistics, they are clearly distinct diseases [24]. Epidemiological and clinical studies have led some authors to suggest that tumors located at the esophageal-gastric junction are distinct from other tumors located in the esophagus or distal stomach, with differing risk factors, tumor characteristics, and biological behavior [25,26]. The association between tumor located at the esophageal-gastric junction and more advanced disease stage has been reported elsewhere and some authors have attributed these findings to more aggressive behavior of junctional tumor [26]. Junctional tumors are associated with adverse prognosis compared with other esophageal and gastric cancers. The anatomical site of these tumors potentially allows tumor spread to lymph nodes located above or below the diaphragm [27]. It is feasible that tumors located in cardia are more aggressive than the ones of distal stomach, and the former ones are usually diagnosed at advanced stage [28,29]. Our study also demonstrated this situation that the 5-years relative survival rate of cardia cancer was the lowest compared with other tumor sites.

One strength of our study was that the databases were acquired from the Shanghai Cancer Registry, the oldest population based cancer registry in mainland China. The registry has contributed data on survival from cancer sites or types registered during 1988–1991 to the first volume of IARC publication on Cancer. Survival data obtained from a population-based cancer registry ideally portrays the average outcome of the disease which avoids the selective bias commonly appears in hospital sourced cases. Comparing to clinical survival study providing information about the treatment, the population-based survival study can evaluate the effectiveness of healthcare systems [30,31]. Besides, population-based cancer registration is necessary to monitor cancer incidence and estimate cancer prevalence [32].

One limitation of this study is that there were 58.4% patients reported with unknown stages. It might be attributed to missing information or patients with unresected cancers. If these cases with unknown stages were gastric cancer patients unable to be surgically resected (so, without TNM stage), usually the survival rate was not far from that of patients with stage IV cases. In this study, their survival was not far from that of stage III, suggesting that most of cases correspond to missing information. Unfortunately, we couldn’t distinguish them based on the current database because all these patients were registered as NOS in the notification cards reported 10 years ago. Secondly, it has been reported that cancer site-related factors may influence the outcome. However, due to the retrospective nature of the present study, we failed to obtain all the needed information for the sites which could have contributed to the bias in estimating the survival rate and thus the influence on the outcome. Thirdly, in the present study, we chose the “classical” relative survival method for cancer survival estimation which may not correctly estimate the net survival, whenever a factor influences jointly the mortality due to cancer or the population life tables used for other-cause mortality. The “net” survival developed by Pohar *et al.* might be an alternative way used in the future to replace the relative survival in population-based studies [33].

## Conclusions

In conclusion, the survival probability of patients with gastric cancer in Shanghai has improved significantly during the last decades. Age, stage and site of tumor have impacts on patient’s prognosis. Information from this study is useful for understanding survival differences that are influenced by changing prevention and treatment strategies.

## Competing interests

All authors disclose no financial and personal relationships with other people or organizations that could inappropriately influence (bias) their work.

## Authors’ contributions

LZ and YZ planned and co-ordinate the study. CW, PX, MZ, LZ, SC, XL, JG and YZ are responsible for the patient follow- up. LZ and MZ drafted the manuscript. CW and YZ were responsible for the statistics. All authors read and approved the final manuscript.

## Pre-publication history

The pre-publication history for this paper can be accessed here:

http://www.biomedcentral.com/1471-2407/14/300/prepub

## Supplementary Material

Additional file 1: Table S1 Gastric cancer cases and study population from 1972 to 2003 in Shanghai, China. Click here for file
