# Supplementary material for: The survival and the long-term trends of patients with gastric cancer in Shanghai, China
Source: BMC Cancer. 2014 Apr 29;14:300. doi: 10.1186/1471-2407-14-300 (PMC4243141; doi:10.1186/1471-2407-14-300)
Supplement: Additional file 1: Table S1 — Gastric cancer cases and study population from 1972 to 2003 in Shanghai, China. [file 1471-2407-14-300-S1.docx]

**Additional file 1: Table S1. Gastric cancer cases and** study population from 1972 to 2003 in Shanghai, China

| Period | Area | Annual population  (million) | Gastric cancer  cases |
| --- | --- | --- | --- |
| 1972-1976 | Urban | 27.88 | 12147 |
| 1980-1984 | Urban | 30.34 | 13991 |
| 1988-1991 | Urban | 28.29 | 12358 |
| 1992-1995 | Urban | 26.82 | 11735 |
| 2002-2003 | Urban | 12.44 | 5139 |
|  | Suburb | 14.20 | 5770 |
